# Serum tissue inhibitor of metalloproteinase‐1 and risk of cognitive impairment after acute ischaemic stroke

**DOI:** 10.1111/jcmm.15369

**Published:** 2020-05-20

**Authors:** Jinzhuo Ge, Ruyi Li, Pengcheng Yuan, Bizhong Che, Xiaoqing Bu, Hancheng Shao, Tan Xu, Zhong Ju, Jintao Zhang, Yonghong Zhang, Chongke Zhong

**Affiliations:** ^1^ Department of Epidemiology School of Public Health and Jiangsu Key Laboratory of Preventive and Translational Medicine for Geriatric Diseases Medical College of Soochow University Suzhou China; ^2^ Department of Epidemiology School of Public Health Chongqing Medical University Chongqing China; ^3^ Department of Neurology Kerqin District First People's Hospital of Tongliao City Inner Mongolia China; ^4^ Department of Neurology The 88th Hospital of PLA Shandong China

**Keywords:** cognitive impairment, extracellular matrix biomarkers, ischaemic stroke, tissue inhibitor of metalloproteinase‐1

## Abstract

The expression of tissue inhibitor metalloproteinase‐1 (TIMP‐1) significantly increased after acute cerebral ischaemia and involved in neurodegeneration. The purpose was to prospectively investigate the relationship between serum TIMP‐1 with post‐stroke cognitive impairment. Our participants were from an ancillary study of China Antihypertensive Trial in Acute Ischemic Stroke. 598 ischaemic stroke patients from seven participating hospitals were included. Cognitive impairment was evaluated using Mini‐Mental State Examination (MMSE) and Montreal Cognitive Assessment (MoCA) at 3 months. 316 (52.84%) or 384 (64.21%) participants had cognitive impairment according to MMSE or MoCA, respectively. Compared with the first quartile of TIMP‐1, the multivariate‐adjusted odds ratios (95% confidence intervals) for the highest quartile were 1.80 (1.09‐2.97) for cognitive impairment defined by MMSE and 2.55 (1.49‐4.35) by MoCA. Multiple‐adjusted spline regression models showed linear associations between TIMP‐1 concentrations and cognitive impairment (*P* value for linearity < 0.01). The addition of TIMP‐1 to models including conventional factors improved reclassification for cognitive impairment, as shown by net reclassification index or integrated discrimination improvement (*P* < 0.05). Participants with both higher TIMP‐1 and matrix metalloproteinase‐9 levels simultaneously had highest risk of cognitive impairment. Higher serum TIMP‐1 levels were associated with increased risk of cognitive impairment after acute ischaemic stroke, independently of established risk factors.

## 1 INTRODUCTION

Stroke is one of the leading cause of death and long‐term disability in the world.^1^ Cognitive impairment is common among stroke survivors, nearly one in three has milder cognitive impairment, and another one in four develops dementia.^2^, ^3^, ^4^ People with cognitive impairment or dementia suffer a lot from physical and mental pain and bring a heavy economic burden. Thus, early identification of high‐risk stroke patients of cognitive impairment is of great importance.

Tissue inhibitor of metalloproteinase‐1 (TIMP‐1) is known as a potent endogenous inhibitor of matrix metalloproteinases‐9 (MMP‐9) and involves in various pathological processes including extracellular matrix degradation, inflammation, fibrosis, apoptosis, differentiation and angiogenesis.^5^, ^6^ Emerging evidences suggest that both TIMP‐1 and MMP‐9 are promising cardiovascular biomarkers.^7^, ^8^ Previous studies indicated that circulating levels of TIMP‐1 significantly increased after acute cerebral ischaemia and played a role in the prognostication of ischaemic stroke.^9^, ^10^ However, the impact of serum TIMP‐1 on subsequent cognitive impairment among patients with ischaemic stroke remains unknown. The expression of TIMP‐1 had been reported to be increased in cerebrospinal fluid (CSF) of neurodegenerative diseases and significantly correlated with Alzheimer's disease (AD) biomarkers.^11^, ^12^, ^13^ Our recent study found that serum MMP‐9 could be a biomarker for predicting post‐stroke cognitive impairment.^14^ We then hypothesized that higher TIMP‐1 levels in the acute phase could increase the risk of post‐stroke cognitive impairment.

Accordingly, we studied the relationship between serum TIMP‐1 and cognitive impairment in patients with acute ischaemic stroke and investigated whether TIMP‐1 provided any predictive value for cognitive impairment. In addition, we also examined the joint effect of serum TIMP‐1 and MMP‐9 on the risk of subsequent cognitive impairment.

## 2 MATERIALS AND METHODS

### 2.1 Participants

The China Antihypertensive Trial in Acute Ischemic Stroke (CATIS) is a multicenter randomized controlled clinical trial designed to test whether early antihypertensive intervention after acute ischaemic stroke would reduce death or major disability.^15^ In brief, CATIS was performed in 26 hospitals across China from August 2009 to May 2013. 4071 patients ≥22 years who had first‐ever ischaemic stroke, confirmed by CT or MRI of the brain within 48 hours of symptom onset, and who had an elevated systolic blood pressure (BP) between 140 to <220 mm Hg were recruited. The present prospective study was based on a pre‐planned ancillary study of CATIS trial, which was designed to examine the effect of early antihypertensive treatment on cognitive function after randomization.^16^

In this ancillary study, ischaemic stroke patients were selected from seven participating hospitals. After excluding patients with severe visual or hearing impairment, we consecutively recruited 660 participants for cognitive function assessment from August 2009 to November 2012. During this period, 15 patients lost to follow‐up and seven patients died. 638 acute ischaemic stroke patients completed the cognitive function assessments. We further excluded 40 participants because they refused to offer samples or we failed to measure TIMP‐1 concentrations, and therefore, 598 patients successfully measured serum TIMP‐1 and were finally involved in our analysis.

Our study was approved by the Institutional Review Boards in Soochow University in China and Tulane University in the United States, as well as ethical committees at the seven participating hospitals. All participants provided written consents.

### 2.2 Assessment of serum TIMP‐1 and covariates

Fasting blood samples were drawn within 24 hours of patients' hospital admission. Blood samples were separated at clinical laboratories of each participating hospitals and immediately frozen at −80°C. Serum TIMP‐1 and MMP‐9 concentrations were determined centrally at Soochow University using a commercially available ELISA kit (R&D Systems, Minneapolis, MN, USA). Intra‐ and inter‐assay coefficients of variation were 3.9% and 4.9% for TIMP‐1 and 2.0% and 6.9% for MMP‐9, respectively. Laboratory technicians who performed biomarkers measurement were blinded to study outcomes. Serum lipids and plasma glucose were obtained at the participating hospitals at admission.

Demographic characteristics, clinical features and medical histories were collected at baseline. Stroke severity was evaluated by the NIH Stroke Scale (NIHSS) score.^17^ BPs were measured with the study participants in a supine position using a standard mercury sphygmomanometer. Three BPs were obtained at baseline according to a standardized protocol.^18^ Hypertension is defined as systolic BP ≥ 140 mm Hg or diastolic BP ≥ 90 mm Hg or on antihypertensive medication before stroke onset. Diabetes mellitus is defined as fasting plasma glucose (FPG) ≥ 126 mg/dL or use of antidiabetic drugs before stroke onset. Those who with total cholesterol ≥ 240 mg/dL, or high‐density lipoprotein cholesterol < 35mg/dL, or use of lipid‐lowering agents before stroke onset were diagnosed with dyslipidemia.

### 2.3 Assessment of outcomes

Our study was cognitive impairment and was evaluated using the Mini‐Mental State Examination (MMSE) and the Montreal Cognitive Assessment (MoCA) by trained neurologists at 3 months.^19^, ^20^ Both MoCA and MMSE have been translated into Chinese and extensive used in assessments of cognitive impairment. The MMSE tests cognitive function in domains including orientation, registration, attention and calculation, language, recall and visual construction.^19^ The MoCA evaluates seven aspects: visuospatial/executive functions, attention, abstraction, memory, language, naming and orientation.^20^ One point was added to total MoCA score (if <30) when ischaemic stroke patients had <12 years of education.^20^ In our study, cognitive impairment was defined as a MMSE score <27^21^, ^22^, ^23^ or a MoCA score <25^22^, ^24^ according to the recommended cut‐offs. In addition, to test the effect of serum TIMP‐1 on severity of cognitive impairment, cognitive function was further categorized as follows: 0‐22 is defined as severe cognitive impairment, 23‐26 as mild cognitive impairment and 27‐30 as no cognitive impairment for MMSE score; 0‐19 is defined as severe cognitive impairment, 20‐24 as mild cognitive impairment and 25‐30 as no cognitive impairment for MoCA scores.^21^, ^22^

### 2.4 Statistical analysis

All study participants were divided into four subgroups based on the quartile of serum TIMP‐1, and baseline characteristics were compared between the different subgroups. The logistic regression models were used to assess the relationship between serum TIMP‐1 and cognitive function. First, odds ratios (ORs) of the risk of cognitive impairment and corresponding 95% confidence intervals (CIs) were calculated by the categorical and ordinal logistic regression model. Three logistic regression models were constructed. In model 1, we adjusted age, sex and education level. Model 2 adjusted the factors included by model 1 and admission NIHSS score, time from onset to randomization, body mass index, systolic BP, current smoking and alcohol drinking. Model 3 adjusted the factors included by model 2 as well as ischaemic stroke subtype, history of hypertension, hyperlipidemia, diabetes mellitus and coronary heart disease, family history of stroke and use of antihypertensive drugs. The selection of covariates was based on previous studies. Second, the median of each group of serum TIMP‐1 values was used as a predictor to test the linear trend among groups. Third, serum TIMP‐1 levels were also logarithm‐transformed, and OR (95% CI) for per 1‐SD increment of logarithm‐transformed TIMP‐1 was calculated. Fourth, a sensitivity analysis was conducted by including randomized treatment in the multivariable model to control the effect of immediate BP reduction during hospitalization.

We used restricted cubic splines regression model to explore the linear relationship between serum TIMP‐1 and cognitive function with four knots (at the 5th, 35th, 65th and 95th percentiles).^25^ Furthermore, net reclassification index (NRI) and integrated discrimination improvement (IDI) are two objective indicators used to quantify the incremental prognostic effect by new biomarkers over an existing prediction model, and we calculated NRI and IDI to assess the predictive power of TIMP‐1 added to conventional risk factor models.^26^ In addition, subgroup analyses were conducted to explore the potential effect modified by age, sex, education, body mass index, admission NIHSS score, smoking, alcohol consumption, history of hypertension and receiving immediate BP reduction. Additionally, the joint effect of extracellular matrix biomarkers on cognitive impairment was investigated, and the cut points of TIMP‐1 and MMP‐9 were obtained from the receiver operating characteristic curves. All *P* values were two tailed, and a significance level of 0.05 was used. Statistical analysis was conducted with SAS statistical software (version 9.4, Cary, NC, USA).

## 3 RESULTS

### 3.1 Baseline characteristics

There were 598 patients (414 men and 184 women; mean age, 59.9 ± 10.5 years) with a median serum TIMP‐1 of 185.7 ng/mL (interquartile range 154.4‐220.6 ng/mL) enrolled in this study. Study participants with a higher serum TIMP‐1 levels tend to be male and alcohol drinkers in comparison with those with lower serum TIMP‐1 levels. However, no obvious difference was observed in this study between groups in terms of age, education, cigarette smoking, time from onset to randomization, systolic BP or diastolic BP and other baseline characteristics (Table 1). Comparison of baseline characteristics between enrolled group and excluded group in our study is shown in Table S1, and most characteristics were comparable between two groups.

**TABLE 1:** Characteristics of participants according to serum TIMP‐1 quartiles

Characteristics^a^	TIMP‐1, ng/mL					*P* Value for trend
	Total	<154.4	154.4‐185.7	185.7‐220.6	≥220.6	
No. of subject	598	148	150	149	151	—
Age, y	59.9 ± 10.5	60.0 ± 10.2	58.7 ± 10.0	60.9 ± 10.6	60.0 ± 11.0	0.58
Male sex	414 (69.2)	93 (62.8)	92 (61.3)	113 (75.8)	116 (76.8)	0.001
Education, y	6.9 ± 3.6	6.8 ± 3.5	6.8 ± 3.5	6.9 ± 3.6	7.0 ± 3.9	0.59
Current cigarette smoking	223 (37.3)	49 (33.1)	58 (38.7)	65 (43.6)	51 (33.8)	0.71
Current alcohol drinking	205 (34.3)	37 (25.0)	51 (34.0)	56 (37.6)	61 (40.4)	0.004
Time from onset to randomization, h	10.8 (5.0‐24.0)	8.6 (4.0‐24.0)	12.0 (6.0‐24.0)	12.0(6.0‐24.0)	9.0 (4.5‐24.0)	0.45
Baseline systolic BP, mm Hg	167.3 ± 16.7	166.5 ± 16.0	167.0 ± 16.7	166.8 ± 14.9	169.0 ± 18.9	0.22
Baseline diastolic BP, mm Hg	98.3 ± 10.1	97.5 ± 9.8	99.1 ± 10.0	97.5 ± 9.2	99.0 ± 11.1	0.44
Body mass index, kg/m^2^	24.9 ± 3.1	24.8 ± 3.1	25.0 ± 3.2	24.9 ± 3.2	25.0 ± 3.0	0.68
Baseline NIHSS score	4.0 (3.0‐7.0)	4.0 (3.0‐8.0)	4.0 (2.0‐7.0)	4.0 (3.0‐7.0)	4.0 (2.0‐7.0)	0.68
History of hypertension	462 (77.3)	114 (77.0)	120 (80.0)	110 (73.8)	118 (78.2)	0.86
Use of antihypertensive drugs	266 (44.5)	64 (43.2)	71 (47.3)	63 (42.3)	68 (45.0)	0.99
History of coronary heart disease	64 (10.7)	16 (10.8)	12 (8.0)	17 (11.4)	19 (12.6)	0.44
History of diabetes mellitus	102 (17.1)	23 (15.5)	24 (16.0)	24 (16.1)	31 (20.5)	0.27
History of hyperlipidemia	42 (7.0)	17 (11.5)	7 (4.7)	9 (6.0)	9 (6.0)	0.11
Family history of stroke	100 (16.7)	29 (19.6)	23 (15.3)	28 (18.8)	20 (13.3)	0.25
Fasting plasma glucose (mmol/L)	6.6 ± 2.7	6.6 ± 2.3	7.0 ± 3.3	6.4 ± 2.3	6.6 ± 2.9	0.53
Total cholesterol (mmol/L)	5.0 ± 1.1	5.0 ± 1.1	5.2 ± 1.1	4.9 ± 1.0	4.9 ± 1.2	0.22
Triglycerides (mmol/L)	1.9 ± 1.3	2.0 ± 1.3	1.9 ± 1.1	1.9 ± 1.5	1.9 ± 1.3	0.55
Low density lipoprotein (mmol/L)	2.9 ± 0.9	2.8 ± 0.9	3.0 ± 1.0	2.8 ± 0.9	2.8 ± 0.9	0.83
High‐density lipoprotein (mmol/L)	1.3 ± 0.5	1.3 ± 0.4	1.3 ± 0.3	1.4 ± 0.7	1.3 ± 0.6	0.54
Ischaemic stroke subtype						0.54
Thrombotic	384 (64.2)	93 (62.8)	99 (66.0)	95 (63.8)	97 (64.2)	
Embolic	22 (3.7)	2 (1.4)	5 (3.3)	4 (2.7)	11 (7.3)	
Lacunar	192 (32.1)	53 (35.8)	46 (30.7)	50 (33.6)	43 (28.5)	

Abbreviations: BP, blood pressure; NIHSS, National Institutes of Health Stroke ScaleTIMP‐1, tissue inhibitor of metalloproteinase‐1.

^a^ Continuous variables are expressed as mean ± SD or as median (interquartile range). Categorical variables are expressed as frequency (percentage).

### 3.2 Serum TIMP‐1 and cognitive impairment

**FIGURE 1:**
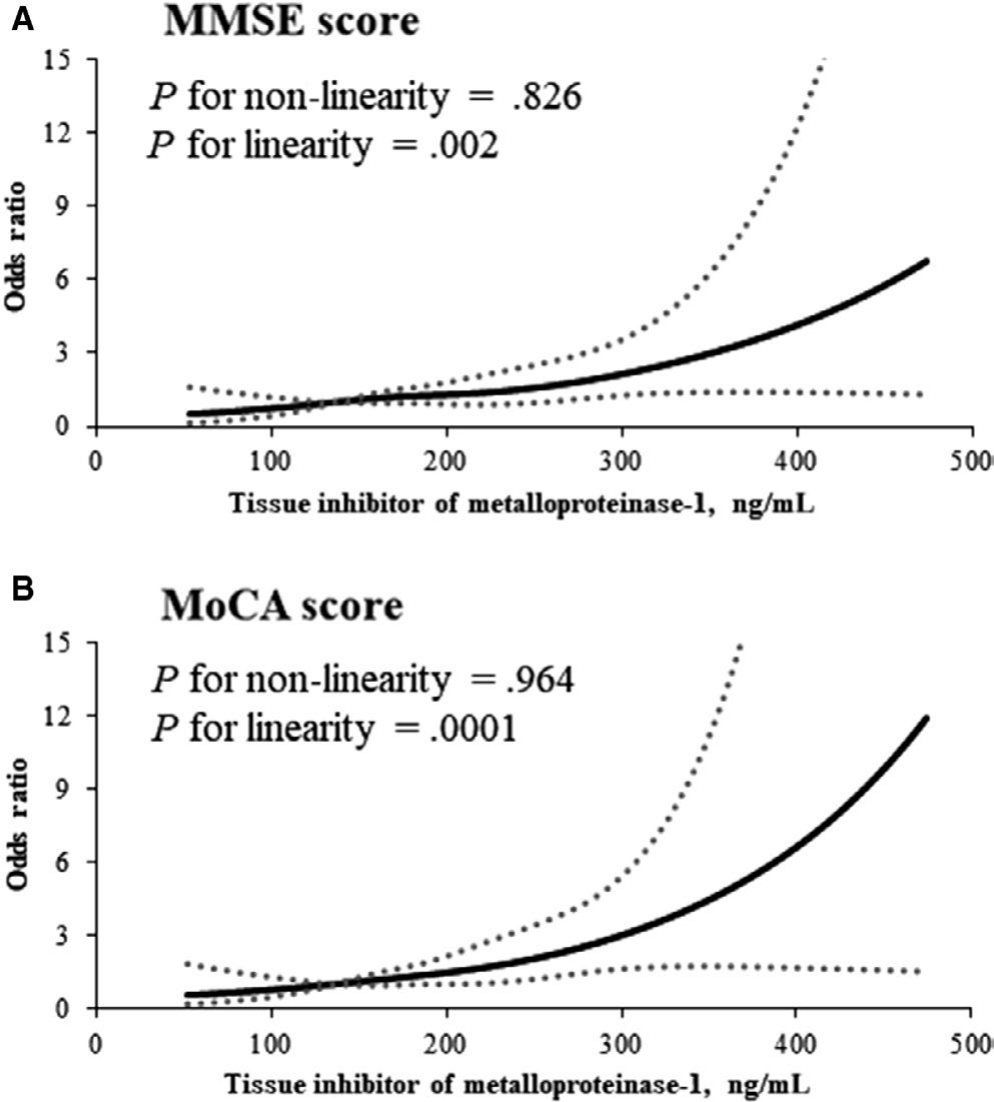
Association of serum TIMP‐1 with risk of cognitive impairment after acute ischaemic stroke. Odds ratios and 95% confidence intervals derived from restricted cubic spline regression, with knots placed at the 5th, 35th, 65th and 95th percentiles of the distribution of serum TIMP‐1. The reference point is the midpoint of the reference group from categorical analysis. Odds ratios were adjusted for the same variables as model 3 in Table 2. Panel A: Mini‐Mental State Examination score of <27; Panel B: Montreal Cognitive Assessment score of <25

A total of 316 (52.84%) or 384 (64.21%) participants had cognitive impairment at 3 months according to MMSE or MoCA, respectively. The rate of cognitive impairment was higher among patients with higher TIMP‐1 levels than that patients with lower TIMP‐1 levels (Table 2). In age, sex and education level‐adjusted logistic models, the ORs of cognitive impairment were significantly higher among participants with TIMP‐1 levels in the highest quartile (≥220.6 ng/mL) compared with those in the lowest quartile (<154.4 ng/mL). After further adjustment for clinical features, medical histories and other covariates in model 3, compared with the lowest quartile of TIMP‐1, the ORs for the highest quartile were 1.80 (95% CI, 1.09‐2.97) for cognitive impairment defined by MMSE and 2.55 (95% CI, 1.49‐4.35) by MoCA. Moreover, the corresponding ORs for each SD increase in logarithm TIMP‐1 were 1.23 (95% CI, 1.01‐1.48) and 1.36 (95% CI, 1.09‐1.70), respectively. In addition, the sensitivity analysis (model 4) suggested that further including randomized treatment in the model 3 did not change the significant association between serum TIMP‐1 and post‐stroke cognitive impairment. Multivariable‐adjusted spline regression models showed linear associations between serum TIMP‐1 and cognitive impairment defined by both MMSE (*P* for linearity = 0.002) and MoCA (*P* for linearity = 0.0001) score (Figure 1). Subgroup analyses showed that the relationship between TIMP‐1 and cognitive impairment was similar across subgroups stratified according to age, sex, body mass index, admission NIHSS score, smoking status, alcohol consumption, history of hypertension and receiving immediate BP reduction (Table S2). However, when cognitive impairment defined by MoCA, we found that education year modified the association between TIMP‐1 and cognitive impairment (*P* = 0.042 for interaction).

**TABLE 2:** ORs and 95% CIs for the risk of cognitive impairment according to TIMP‐1 quartiles

Variable	TIMP‐1, ng/mL				*P* Value for Trend	Each SD (0.17 ng/mL) Increase in Logarithm TIMP‐1
	<154.4	154.4‐185.7	185.7‐220.6	≥220.6		
Median	136.7	168.2	199.5	259.9	—	—
Cognitive Impairment: MMSE score						
Events, n (%)	73 (49.32)	69 (46.00)	82 (55.03)	92 (60.93)	—	316 (52.84)
Model 1	1.00	0.94 (0.58‐1.50)	1.20 (0.75‐1.92)	1.62 (1.00‐2.61)	0.02	1.17 (0.98‐1.41)
Model 2	1.00	0.98 (0.61‐1.58)	1.24 (0.77‐2.02)	1.79 (1.10‐2.93)	0.009	1.21 (1.00‐1.46)
Model 3	1.00	0.98 (0.60‐1.59)	1.26 (0.77‐2.07)	1.80 (1.09‐2.97)	0.01	1.23 (1.01‐1.48)
Model 4	1.00	0.97 (0.60‐1.59)	1.25 (0.77‐2.07)	1.79 (1.08‐2.96)	0.01	1.23 (1.01‐1.48)
Cognitive Impairment: MoCA score						
Events, n (%)	85 (57.43)	87 (58.00)	99 (66.44)	113 (74.83)	—	384 (64.21)
Model 1	1.00	1.09 (0.68‐1.75)	1.47 (0.90‐2.39)	2.35 (1.41‐3.91)	<0.001	1.32 (1.07‐1.62)
Model 2	1.00	1.13 (0.69‐1.83)	1.51 (0.91‐2.49)	2.65 (1.56‐4.49)	<0.001	1.36 (1.10‐1.70)
Model 3	1.00	1.08 (0.66‐1.77)	1.50 (0.90‐2.49)	2.55 (1.49‐4.35)	<0.001	1.36 (1.09‐1.70)
Model 4	1.00	1.08 (0.66‐1.77)	1.50 (0.90‐2.50)	2.55 (1.49‐4.36)	<0.001	1.36 (1.09‐1.70)

Note

Model 1, adjusted for age, sex and education level.

Model 2, adjusted for model 1 and further adjusted for time from onset to randomization, systolic blood pressure, baseline National Institutes of Health Stroke Scale scores, body mass index, current smoking and alcohol drinking.

Model 3, adjusted for model 2 and further adjusted for medical history (hypertension, diabetes mellitus, hyperlipidemia and coronary heart disease), family history of stroke, use of antihypertensive medications and ischaemic stroke subtype.

Model 4 (sensitivity analysis), adjusted for model 3 and further adjusted for randomized treatment.

MMSE score of <27 or MoCA score of <25 indicates cognitive impairment.

Abbreviations: CI, confidence interval; MMSE, Mini‐Mental State Examination; MoCA, Montreal Cognitive Assessment; OR, odds ratio; TIMP‐1, tissue inhibitor of metalloproteinase‐1.

**FIGURE 2:**
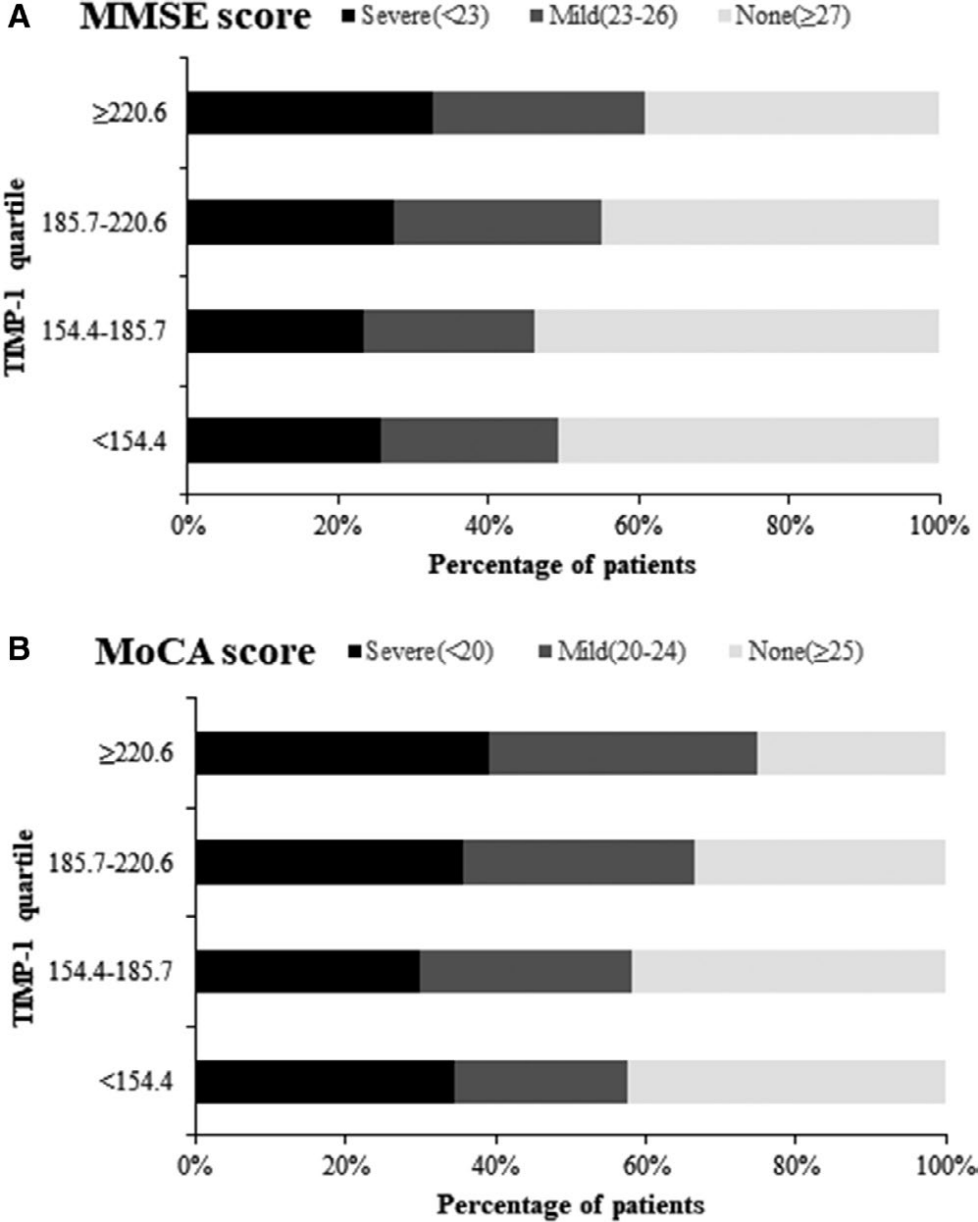
Serum TIMP‐1 and cognitive impairment severity. Adjusted odds ratio of ordinal logistic regression analysis for highest vs lowest quartile of serum TIMP‐1:1.58 (95% confidence interval, 1.01‐2.48; *P* trend = 0.020) for Mini‐Mental State Examination (A); and 1.67 (95% confidence interval, 1.07‐2.61; *P* trend = 0.008) for Montreal Cognitive Assessment (B)

Participants were further divided into three groups according to the severity of cognitive impairment. And the ordinal analysis suggested significant associations between serum TIMP‐1 and cognitive impairment severity, the ORs were 1.58 (95% CI, 1.01‐2.48; *P* trend = 0.020) for MMSE and 1.67 (95% CI, 1.07‐2.61; *P* trend = 0.008) for MoCA, when two extreme quartiles were compared (Figure 2).

### 3.3 Reclassification of serum TIMP‐1

We examined the risk reclassification value of TIMP‐1 by adding serum TIMP‐1 to conventional factors models (Table 3). The conventional model included all covariates in Model 3. Adding serum TIMP‐1 to the conventional models improved risk reclassification for cognitive impairment, as evidenced by an increase in NRI of 0.191 (95% CI, 0.034‐0.349; *P* = 0.020), IDI of 0.012 (95% CI, 0.004‐0.021; *P* = 0.005) for cognitive impairment was defined by MMSE; and NRI of 0.273 (95% CI, 0.109‐0.437; *P* = 0.001), IDI of 0.026 (95% CI, 0.013‐0.039; *P* < 0.001) for MoCA.

**TABLE 3:** Reclassification statistics (95% CI) for cognitive impairment by serum TIMP‐1 among patients with acute ischaemic stroke

Variable	NRI (Category Free)		IDI	
	Estimate (95% CI)	*P* value	Estimate (95% CI)	*P* value
Cognitive impairment: MMSE score				
Conventional model				
Conventional model + TIMP‐1 (continuous)	0.191 (0.034‐0.349)	0.020	0.012 (0.004‐0.021)	0.005
Conventional model + TIMP‐1 (quartiles)	0.174 (0.015‐0.334)	0.033	0.009 (0.001‐0.017)	0.021
Cognitive impairment: MoCA score				
Conventional model				
Conventional model + TIMP‐1 (continuous)	0.273 (0.109‐0.437)	0.001	0.026 (0.013‐0.039)	<0.001
Conventional model + TIMP‐1 (quartiles)	0.255 (0.089‐0.421)	0.003	0.020 (0.009‐0.032)	<0.001

Note

Conventional model included age, sex, education level, time from onset to randomization, systolic blood pressure, baseline National Institutes of Health Stroke Scale scores, body mass index, current smoking, alcohol drinking, medical history (hypertension, diabetes mellitus, hyperlipidemia and coronary heart disease), family history of stroke, use of antihypertensive medications and ischaemic stroke subtype.

MMSE score of <27 or MoCA score of <25 indicates cognitive impairment.

Abbreviations: CI, confidence interval; IDI, integrated discrimination index; MMSE, Mini‐Mental State Examination; MoCA, Montreal Cognitive Assessment; NRI, net reclassification improvement; TIMP‐1, tissue inhibitor of metalloproteinase‐1.

### 3.4 Joint effect of extracellular matrix biomarkers

Our analysis found that ischaemic patients with both higher serum TIMP‐1 (≥184.2 ng/mL) and MMP‐9 (≥462.6 ng/mL) levels had higher risk of post‐stroke cognitive impairment. Compared with ischaemic stroke patients with both lower serum TIMP‐1 and MMP‐9 levels, the ORs were 2.83 (95% CI, 1.74‐4.60) for cognitive impairment defined by MMSE and 2.99 (95% CI, 1.81‐4.95) by MoCA in participants with both higher levels (Table 4).

**TABLE 4:** Joint effects of serum TIMP‐1 and matrix metalloproteinase‐9 (MMP‐9) on the risk of cognitive impairment after acute ischaemic stroke. The forest plot is misaligned with the Numbers (OR (95% CI))

Timp‐1 ≥184.2 ng/mL	MMP‐9 ≥462.6 ng/mL	Events No (%)	OR (95% CI)	
Cognitive impairment: MMSE score			
−	−	57(40.4)	Reference
+	−	39(50.7)	1.34(0.72‐2.48)
−	+	77(53.9)	1.95(1.17‐3.25)
+	+	123(62.4)	2.83(1.74‐4.60)
Cognitive impairment: MoCA score			
−	−	73(51.8)	Reference
+	−	48(62.3)	1.41(0.75‐2.64)
−	+	89(62.2)	1.72(1.03‐2.89)
+	+	144(73.1)	2.99(1.81‐4.95)

Note

Optimal cut points for TIMP‐1 and MMP‐9 were obtained from the ROC curves.

OR was adjusted for age, sex, education level, time from onset to randomization, systolic blood pressure, baseline National Institutes of Health Stroke Scale scores, body mass index, current smoking, alcohol drinking, medical history (hypertension, diabetes mellitus, hyperlipidemia and coronary heart disease), family history of stroke, use of antihypertensive medications and ischaemic stroke subtype.

Abbreviations: CI, confidence interval; OR, odds ratio.

## 4 DISCUSSION

In the present prospective study, we found significant relationships between serum TIMP‐1 levels and the risk of cognitive impairment, independently of age, education levels, medical history and other confounders. Importantly, including serum TIMP‐1 to the traditional factors models significantly improved the predictive value of post‐stroke cognitive impairment. To our knowledge, this study is the first to prospectively evaluate the association between serum TIMP‐1 levels and subsequent cognitive impairment after acute ischaemic stroke.

Previous studies indicated that TIMP‐1 was associated with the progression of atherosclerosis could serve as a potential prognostic biomarker of cardiovascular diseases and mortality in several pathophysiological conditions including myocardial infarction and coronary artery diseases.^27^, ^28^, ^29^ Moreover, circulating TIMP‐1 and MMP‐9 had been reported to be increased in stroke, multiple sclerosis and other neurological diseases.^9^, ^12^ Both TIMP‐1 and MMP‐9 were suggested to have important role in the prognostication of ischaemic stroke,^10^, ^30^ and the coexistence of higher serum TIMP‐1 and MMP‐9 was associated with highest risk of adverse clinical outcomes after ischaemic stroke.^31^

Studies evaluating the associations between TIMP‐1 levels and risk of cognitive impairment after ischaemic stroke are sparse. Aberrant expression of TIMP‐1 was observed in brain tissue, CSF and plasma of neurodegenerative diseases.^12^, ^32^ For example, Hanzel et al^32^ found that TIMP‐1 levels in CSF of patients with mild cognitive impairment were higher than the subjective cognitive impairment patients. Lorenzl et al^12^ measured the levels of TIMP‐1 in CSF samples of patients with Parkinson's Disease, AD, Huntington's Disease and amyotrophic lateral sclerosis, and demonstrated that elevated TIMP‐1 levels were observed in all participants. They hypothesized that TIMP‐1 played a potential role in neurodegenerative diseases. Furthermore, Bjerke et al^13^ found that in patients with AD, TIMP‐1 in the CSF was correlated with the concentrations of commonly used biomarkers of AD such as tau protein. All these studies suggested a potential role of TIMP‐1 in the development of cognitive impairment. Here, our prospective study verified this hypothesis, serum TIMP‐1 was associated with subsequent cognitive impairment among acute ischaemic stroke patients, and provided additional predictive value beyond established risk factors for cognitive impairment. Our recent study showed that serum MMP‐9 could be a potential prognostic factor for 3‐month cognitive impairment.^14^ Interestingly, we observed a joint effect of serum TIMP‐1 and MMP‐9 on post‐stroke cognitive impairment. Taken together, these findings provide valuable insight into extracellular matrix dysfunction in the prediction of cognitive impairment. Previous studies suggested that dietary interventions or pharmaceutical inhibitors could influence extracellular matrix remodelling,^33^ which might reduce the damage to the blood‐brain barrier and maintain the normal function of the central nervous system. Future clinical trials are needed to test the effect of relief from extracellular matrix dysfunction on cognitive impairment in acute ischaemic stroke patients.

The mechanisms underlying the relationship of serum TIMP‐1 with cognitive impairment so far is unclear, but previous studies have provided some physiological pathways. First, higher MMP‐9 is involved in the neuroinflammation and blood‐brain barrier damage and correlated with post‐stroke cognitive impairment.^14^, ^34^ Increased serum TIMP‐1 may be an adaptive physiological response to enhanced MMP‐9 activity, in order to regulate excessive proteolytic activity and maintain extracellular matrix homeostasis in the acute phase ischaemic stroke. Moreover, independent of the MMP inhibitory property, TIMP‐1 also exhibits other biological actions, including promoting cell growth, apoptosis, anti‐angiogenesis, oncogenesis and neuroinflammation.^11^ Furthermore, TIMP‐1 was reported to be associated with white matter hyperintensities and low brain volume, which could increase risk of the development of ischaemic stroke and mild cognitive impairment.^35^, ^36^ TIMP‐1 is produced by neurons, microglia, astrocytes, or endothelial cells, and regulates the remodelling of central nervous system extracellular matrices together with MMP‐9.^37^, ^38^ Increased TIMP‐1 and MMP‐9 levels may disrupt the microvascular basal layer and lead to leukoaraiosis, which in turn causes cognitive impairment and dementia.^35^ In addition, TIMP‐1 concentrations were found to be related to left ventricular hypertrophy (LVH) and systolic dysfunctions,^39^ while LVH was independently associated with cognitive decline or dementia.^40^

Our study has several strengths. First, this prospective clinical study came from a randomized subset of the CATIS trial, rigid quality control procedures and standardized protocols were used for data collection. Second, we conducted a comprehensive and systematic collection of established risk factors, and after adjusting for these covariate variables, the significant findings remain. Third, two evaluations scales were used for cognitive impairment assessment, the consistent findings based on MMSE and MoCA indicated the robustness of the relationship between serum TIMP‐1 and cognitive impairment. Another important advantage is that we measured TIMP‐1 concentrations from serum sample, which is more readily available than CSF or brain tissue. This advantage provides us a convenient opportunity to study the prognostic utility of TIMP‐1 for post‐stroke cognitive impairment.

Some limitations need to be considered. First, our study was from the CATIS trial, the study excluded people with BP ≥ 220/120 mm Hg at admission or received intravenous thrombolytic therapy, which may cause selection bias. However, it is reported that few ischaemic patients have BP ≥ 220/120 mm Hg and receive intravenous thrombolytic therapy in China. Nevertheless, these limited the generalizability of our findings to all patients with acute ischaemic stroke. Second, all our study participants were from China, so it should be cautiously when the findings extrapolate to other populations. Third, we did not collect dynamic changes of TIMP‐1 levels before and after ischaemic stroke in the population. This limited us to explore the association between TIMP‐1 changes and cognitive impairment.

## 5 CONCLUSIONS

Higher serum TIMP‐1 levels were associated with increased risk of cognitive impairment at 3 months after acute ischaemic stroke, independently of established risk factors. Our study provided further evidence supporting the important role of extracellular matrix biomarkers in the prediction of post‐stroke cognitive impairment.

## CONFLICT OF INTEREST

The authors confirm that there are no conflicts of interest.

## AUTHOR CONTRIBUTION

Each author has made an important scientific contribution to this study and has assisted with the drafting or revising of the manuscript.

## Supporting information

S1‐S2

## Data Availability

The data are free access to available upon request.
