# Supplementary material for: Serum tissue inhibitor of metalloproteinase‐1 and risk of cognitive impairment after acute ischaemic stroke
Source: J Cell Mol Med. 2020 May 20;24(13):7470–8. doi: 10.1111/jcmm.15369 (PMC7339163; doi:10.1111/jcmm.15369)
Supplement: Supplementary file 1 — S1‐S2 [file JCMM-24-7470-s001.docx]

Table S1. Baseline characteristics of acute ischemic stroke patients.

| Characteristics | Excluded | Enrolled | *P* value |
| --- | --- | --- | --- |
| Number of patients | 3473 | 598 | . . . |
| Age, y | 62.3±10.9 | 59.9±10.5 | 0.2482 |
| Male sex | 2190(63.1) | 414(69.2) | 0.0037 |
| Education, y | 6.2±3.7 | 6.9±3.6 | <0.0001 |
| Current cigarette smoking | 1262(36.3) | 223(37.3) | 0.6546 |
| Current alcohol drinking | 1048(30.2) | 205(34.3) | 0.0446 |
| Time from onset to randomization, h | 10.0(4.5-24.0) | 10.8(5.0-24.0) | 0.8131 |
| Baseline systolic BP, mm H | 165.9±16.9 | 167.3±16.7 | 0. 0384 |
| Baseline diastolic BP, mm Hg | 96.4±11.3 | 98.3±10.06 | 0. 2667 |
| Body mass index, kg/m2 | 25.0±3.2 | 24.9±3.1 | 0. 9999 |
| Baseline NIHSS score | 4.0(2.0-8.0) | 4.0(3.0-7.0) | 0.0671 |
| History of hypertension | 2747(79.1) | 462(77.3) | 0.3095 |
| History of hyperlipidemia | 235(6.8) | 42(7.0) | 0.8177 |
| History of diabetes mellitus | 617(17.8) | 102(17.1) | 0.6746 |
| History of coronary heart disease | 380(10.9) | 64(10.7) | 0.8624 |
| Family history of stroke | 653(18.8) | 100(16.7) | 0.2263 |

BP indicates blood pressure; NIHSS, National Institute of Health Stroke Scale.

Table S2. Subgroup analyses of the association between serum TIMP-1 and cognitive impairment.

| Subgroup | Cognitive impairment (MMSE score) | | | Cognitive impairment (MoCA score) | | |
| --- | --- | --- | --- | --- | --- | --- |
|  | OR (95%CI) |  | P-interaction | OR (95%CI) |  | P-interaction |
| Age, y | |  | 0.6085 |  |  | 0.7945 |
| <65 | 1.72 (0.94-3.15) |  |  | 1.87 (1.00-3.50) |  |  |
| ≥65 | 1.98 (0.74-5.25) |  |  | 7.43 (2.17-25.44) |  |  |
| Sex | |  | 0.4141 |  |  | 0.2315 |
| Male | 1.72 (0.95-3.12) |  |  | 1.92 (1.03-3.60) |  |  |
| Female | 2.90 (0.99-8.49) |  |  | 7.91 (2.08-30.10) |  |  |
| Education | |  | 0.5455 |  |  | 0.0415 |
| <8 | 2.57 (1.23-5.38) |  |  | 4.18 (1.95-8.97) |  |  |
| ≥8 | 1.23 (0.59-2.58) |  |  | 1.49 (0.65-3.43) |  |  |
| BMI | |  | 0.4348 |  |  | 0.8873 |
| <24 | 1.49 (0.59-3.75) |  |  | 4.05 (1.50-10.91) |  |  |
| ≥24 | 2.01 (1.07-3.78) |  |  | 2.07 (1.06-4.02) |  |  |
| Admission NIHSS score | |  | 0.3533 |  |  | 0.6828 |
| <4 | 1.93 (0.84-4.44) |  |  | 2.41 (1.02-5.70) |  |  |
| ≥4 | 1.66 (0.87-3.16) |  |  | 2.61 (1.28-5.29) |  |  |
| Smoking status | |  | 0.4555 |  |  | 0.3622 |
| No | 2.33 (1.24-4.38) |  |  | 3.42 (1.73-6.77) |  |  |
| Yes | 1.27 (0.52-3.12) |  |  | 1.57 (0.61-4.05) |  |  |
| Alcohol consumption | |  | 0.4319 |  |  | 0.8716 |
| No | 2.11 (1.11-4.00) |  |  | 2.85 (1.45-5.62) |  |  |
| Yes | 1.05 (0.42-2.62) |  |  | 1.84 (0.67-5.04) |  |  |
| History of hypertension | |  | 0.2897 |  |  | 0.6928 |
| No | 0.86 (0.27-2.79) |  |  | 1.36 (0.40-4.63) |  |  |
| Yes | 2.21 (1.25-3.92) |  |  | 2.93 (1.59-5.40) |  |  |
| Receiving immediate BP reduction | |  | 0.7098 |  |  | 0.2654 |
| No | 1.37 (0.60-2.71) |  |  | 2.90 (1.28-6.57) |  |  |
| Yes | 2.55 (1.24-5.28) |  | 0.6085 | 2.23 (1.04-4.78) |  | 0.7945 |
|  |  |  |  |  |  |  |

ORs and 95%CIs were calculated for the highest quartile compared to the lowest quartile after adjustment for the same variables as model 3 in Table 2, except for the stratified variable.

Mini-Mental State Examination (MMSE) score of <27 or Montreal Cognitive Assessment (MoCA) score of <25 indicate cognitive impairment.

TIMP-1, tissue inhibitor of metalloproteinase-1; OR, odds ratio; CI, confidence interval; NIHSS, National Institutes of Health Stroke Scale; and BP, blood pressure.
